# Rapid, sensitive, and visual detection of mandarin fish ranavirus and infectious spleen and kidney necrosis virus using an RPA-CRISPR/Cas12a system

**DOI:** 10.3389/fmicb.2024.1495777

**Published:** 2024-12-13

**Authors:** Zhoutao Lu, Mincong Liang, Chuanrui Li, Yichun Xu, Shaoping Weng, Jianguo He, Changjun Guo

**Affiliations:** School of Marine Sciences, State Key Laboratory for Biocontrol, Southern Marine Science and Engineering Guangdong Laboratory (Zhuhai), Guangdong Province Key Laboratory for Aquatic Economic Animals and Guangdong Provincial Observation and Research Station for Marine Ranching of the Lingdingyang Bay, Sun Yat-sen University, Guangzhou, China

**Keywords:** iridovirus, RPA, CRISPR/Cas12a, ISKNV, ranavirus

## Abstract

Iridoviruses are large cytoplasmic icosahedral viruses that contain dsDNA. Among them, mandarin fish ranavirus (MRV) and infectious spleen and kidney necrosis virus (ISKNV) are particularly notable due to their high contagiousness and pathogenicity. These viruses pose a significant threat to fish aquaculture, resulting in substantial annual economic losses for the fish farming industry. Therefore, the development of novel, rapid virus detection technologies is essential for the prevention and control of ISKNV and MRV diseases. In this study, we developed a rapid, sensitive, and visual detection method for MRV and ISKNV using the recombinase polymerase amplification (RPA)−CRISPR/Cas12a system. This method can detect as low as 1 copy/μL of MRV and 0.1 copy/μL of ISKNV, demonstrating excellent specificity and reproducibility. The detection can be performed at a constant temperature of 37–39°C, eliminating the need for complex equipment. A 30-min RPA amplification followed by a 15-min CRISPR/Cas reaction is sufficient for detecting most samples. For low-concentration samples, extending the CRISPR/Cas reaction time to 60 min improves result visibility. The designed RPA reaction system is capable of performing reverse transcription of RNA, allowing for the detection of mRNA transcribed from the MCP gene of MRV and ISKNV in the sample. Furthermore, two probes were identified that can be observed without the need for excitation light. In conclusion, a field-suitable detection method for ISKNV and MRV has been established, providing a powerful tool for the prompt diagnosis of these aquatic pathogens and aiding in the prevention and control of ISKNV and MRV diseases.

## 1 Introduction

Iridoviruses are large icosahedral viruses that contain cytoplasmic dsDNA (Chinchar et al., 2017). According to the International Committee on Taxonomy of Viruses (ICTV), the family *Iridoviridae* is primarily divided into two subfamilies: *Alphairidovirinae* and *Betairidovirinae*, which together encompass seven genera (Qin et al., 2023) and 22 recognized species.^1^ Iridoviruses are significant causative agents of outbreaks in cultured fish, and they are among the most widespread and pathogenic viral pathogens affecting farmed fish species (Leiva-Rebollo et al., 2024). Notably, the mandarin fish ranavirus (MRV) and the infectious spleen and kidney necrosis virus (ISKNV) are noteworthy due to their endemicity and high pathogenicity, posing a significant threat to aquaculture. These viruses annually cause economic losses exceeding $1 billion to the fish farming industry. Susceptible species include mandarin fish (*Siniperca chuatsi*), largemouth bass (*Micropterus salmoides*), large yellow croaker (*Pseudosciaena crocea*), and others (He et al., 1998; Grizzle et al., 2002; Wang et al., 2007). Currently, methods such as nested PCR, real-time PCR, and microscopic examination are typically used for the detection and identification of MRV and ISKNV (Fu et al., 2020; Cao et al., 2024; Zhao et al., 2023). Nested PCR, which requires two rounds of PCR amplification followed by gel electrophoresis for visualization, is a time-consuming process (Fu et al., 2020). Although real-time PCR is more rapid and sensitive, it requires expensive equipment, making it unsuitable for on-site detection (Zhao et al., 2023). Conventional light microscopy can only provide preliminary indications of viral infection, while definitive identification of the virus type usually requires the use of purified virus particles and electron microscopy for detailed structural analysis (Zhao et al., 2023). Therefore, the development of novel, rapid virus detection technologies is crucial for preventing and controlling MRV and ISKNV diseases.

Recombinase polymerase amplification (RPA) technology, developed by Piepenburg et al. in 2006 (Piepenburg et al., 2006), is a nucleic acid amplification technique that primarily relies on components derived from the T4 bacteriophage, including recombinase, single-strand binding protein (SSB), and strand-displacing DNA polymerase (Piepenburg et al., 2006). The mechanism of RPA mimics the T4 phage’s DNA replication process, where the recombinase binds to the primer-template complex, enabling homology search and strand invasion (Yonesaki et al., 1985; Shibata et al., 1979; Formosa and Alberts, 1986). Once the complex is positioned on the template, it initiates a strand exchange reaction. SSBs then bind to the displaced DNA strand, stabilizing the formed D-loop structure and preventing the primer from dissociating (Harris and Griffith, 1988). After the recombinase dissociates, the 3′ end of the primer is exposed and recognized by the DNA polymerase, which then adds the corresponding bases according to the template sequence, initiating DNA amplification. The strand-displacing DNA polymerase continues to unwind the double-stranded DNA template while extending the primer, allowing the DNA synthesis process to proceed (Okazaki and Kornberg, 1964). RPA technology, characterized by its simplicity, high sensitivity, extremely rapid amplification, and the ability to operate at a low constant temperature, has been developed for the detection of various pathogens and genes. However, RPA technology has some limitations. Typically, the amplified products need to be purified after amplification; otherwise, it is difficult to obtain ideal bands in agarose gel electrophoresis or lateral flow strip tests. Therefore, achieving convenient detection of the amplified products is crucial (Lobato and O'Sullivan, 2018).

The CRISPR/Cas system comprises clustered regularly interspaced short palindromic repeats (CRISPR) and their associated Cas proteins (Jansen et al., 2002). This system has found widespread application in areas such as cellular imaging, expression regulation, gene editing, and genetic diagnostics (Chen et al., 2013; Zhang et al., 2017; Mali et al., 2013; Joung et al., 2017). Recent discoveries have revealed that Cas12 and Cas13 proteins, guided by CRISPR RNA (crRNA), exhibit both cis-cleavage activity for recognizing and cutting specific targets and trans-cleavage activity that can be activated by the target (Chen et al., 2018; Li et al., 2018; Abudayyeh et al., 2016). This means that, upon specific recognition of the target sequence by Cas proteins and crRNA, they can be activated to non-specifically cleave single-stranded DNA (ssDNA) or single-stranded RNA (ssRNA). Based on this principle, trans-cleavage substrates can be designed as suitable reporter probes. When Cas proteins are activated, these probes are cleaved, releasing a signal that can be used for the detection of target nucleic acids (Chen et al., 2018; Kellner et al., 2019; Li et al., 2018; Li et al., 2019; Gootenberg et al., 2017). Thus, detection systems based on nucleic acid amplification and CRISPR/Cas technology have emerged. In 2017, Zhang Feng et al. combines RPA amplification with Cas13 protein to develop a nucleic acid detection method named SHERLOCK, which can achieve single-molecule detection and distinguish single-nucleotide mismatches (Gootenberg et al., 2017). Meanwhile, Doudna et al. and Wang Jin et al. separately combines isothermal amplification and PCR amplification with Cas12a protein to develop new detection methods. These methods can rapidly and easily identify human papilloma virus subtypes (Chen et al., 2018) and detect human single nucleotide polymorphism genotypes (Li et al., 2018). Beyond applications in human disease and genotype detection, this detection technology has been widely applied in various fields such as agriculture, animal husbandry, and aquaculture for the detection of numerous pathogens. Examples include potato virus Y (He et al., 2022), African swine fever virus (ASFV) (Mao et al., 2023), grouper nervous necrosis virus (Huang et al., 2022), and infectious hematopoietic necrosis virus (Rong et al., 2024). Their excellent detection capabilities are of great significance for disease prevention.

In this study, we achieved a rapid, sensitive, and visual detection of MRV and ISKNV using the RPA-CRISPR/Cas system. This approach offers a potent tool for the prompt diagnosis of these aquatic pathogens, aiding in the prevention and control of MRV and ISKNV diseases.

## 2 Materials and methods

### 2.1 Cells and viruses

Mandarin fish fry (MFF-1) cells were cultured in Dulbecco’s Modified Eagle’s Medium (DMEM; Hyclone, United States) supplemented with 10% fetal bovine serum (FBS; HyClone, United States) and incubated at 27°C in a humidified incubator with a 5% CO_2_ atmosphere (Dong et al., 2008). ISKNV strain NH-2005 (GenBank: OP896201.1), MRV strain NH-1609 (GenBank: MG941005.3), and *Siniperca chuatsi* rhabdovirus (SCRV) strain NH-2103 (GenBank: PQ066876.1) were isolated and stored in our laboratory as previously described (Liang et al., 2024; Qin et al., 2022; Li et al., 2024). Cells were infected with the respective viruses at a multiplicity of infection (MOI) of 1. After 4 h of adsorption, the inoculum was removed, and the cells were washed twice with PBS. Fresh DMEM with 10% FBS was then added as previously described (He et al., 2022). All the cells were harvested at 72 h post-infection for viral nucleic acids extraction.

### 2.2 Extraction of viral nucleic acids

Viral genomic DNA was extracted according to the instructions provided with the FastPure Cell/Tissue DNA Isolation Mini Kit (Vazyme, China). Total RNA was extracted following the protocol of the Eastep^®^ Super Total RNA Extraction Kit (Promega, United States) as previously described (Zhi-Min et al., 2024).

### 2.3 Preparation of standard plasmids

The major capsid protein (MCP) genes of both MRV and ISKNV were selected as targets, and primers were designed to amplify the full length of these genes, as listed in Table 1. After amplifying the target fragments using viral nucleic acid as a template, the pCMV-HA-C vector was linearized using Sal I and Kpn I restriction enzymes (Takara, Japan). Then, the amplified fragment was recombined with the linearized pCMV-HA-C vector using the 2× Ezmax Universal CloneMix kit (Tolobio, China). The ligation products were then transformed into competent cells, and the transformation plates were incubated overnight at 37°C. Single colonies were picked and sent for sequencing to Guangzhou Tianyi Huiyuan Gene Technology Co., Ltd. Colonies with correct sequencing results were selected for large-scale culture, and plasmids were then extracted using the FastPure EndoFree Plasmid Maxi Kit (Vazyme, China). The concentration of the recombinant plasmids was determined using a Nanodrop 2000 ultramicro UV spectrophotometer. Based on the plasmid concentration, the copy number was calculated, and the plasmids were diluted in a tenfold series down to 10^−1^ copies/μL as previously described (Luo et al., 2023). These dilutions were stored at −20°C for future use, while the original plasmid stocks were kept at −80°C for long-term storage.

**Table 1:** PCR primers and sequences.

Primers	Sequence (5′−3′)
MRV-MCP-pCMV-HA-C-F	GGCCCAGGCCCGAATTCGGTCGACATGTCTTCTGTTACGGGTTCTGGC
MRV-MCP-pCMV-HA-C-R	AACATCGTATGGGTAGCCGGTACCTTACAGGATGGGGAAACCCATGG
ISKNV-MCP-pCMV-HA-C-F	GGCCCAGGCCCGAATTCGGTCGACATGTCTGCAATCTCAGGTGC
ISKNV-MCP-pCMV-HA-C-R	AACATCGTATGGGTAGCCGGTACCTTACAGGATAGGGAAGCCTGC

### 2.4 Design and screening of RPA primers

The reference sequences of the *MCP* genes for MRV and ISKNV were downloaded from the NCBI database. Primers were designed using SnapGene v6.0 software. For each virus, three forward and three reverse primers were created, resulting in a total of nine primer pairs that could be combined in various configurations. The specific primer sequences are detailed in Table 2. RPA reactions were carried out using the RT Basic Nucleic Acid Amplification Kit (Lesunbio, China). After completing the 20-min RPA reactions at 39°C, DNA was extracted using phenol-chloroform extraction, and the extracted DNA was analyzed using agarose gel electrophoresis.

**Table 2:** PCR primers and sequences.

Primers	Sequence (5′−3′)
MRV-RPA-F1	CGAGCGTCAGGCTATGAGCAGCTCAGTCAGG
MRV-RPA-F2	AGTCAGGGACATGGTGGTGGAGCAGATGCAG
MRV-RPA-F3	TCCGGTCCACATGGTCAACCCCAAGAACGCC
MRV-RPA-R1	CAACAGGAGTGACGCAAGTGTAGTTGGAACC
MRV-RPA-R2	CGCTCCAACAACAGGAGTGACGCAAGTGTAG
MRV-RPA-R3	CTCCAGGACGGTGTTACCCGCTCCAACAACA
ISKNV-RPA-F1	CGCTGTACTGACAAGCGAGGAGCGTGAGGTG
ISKNV-RPA-F2	CAGTCTAGTCGTAGCATGCTCATTGAACAGT
ISKNV-RPA-F3	GCGTGAGGTGGTGGCCCAGTCTAGTCGTAGC
ISKNV-RPA-R1	CCTTGTTGTTGACATACACGGGACTGGCCGC
ISKNV-RPA-R2	CAATGGCAGATTCACCTTGTTGTTGACATAC
ISKNV-RPA-R3	CCTCGGACAGGGGATTGGTGGCCATCAATGG

### 2.5 Design and synthesis of crRNA

Regions within the RPA products that contain protospacer adjacent motif (PAM) sequences were selected as targets for crRNA design, based on the activation principle of the CRISPR/Cas12a protein. The specific crRNA sequences are detailed in Table 3. We used the Cas12a High Yield crRNA Synthesis and Purification Kit (Tolobio, China) for *in vitro* transcription and purification of the crRNA. The concentration of the purified crRNA was measured using a Nanodrop 2000 ultramicro UV spectrophotometer, and the copy number was calculated using SnapGene v6.0 software. The product was then diluted with diethyl pyrocarbonate (DEPC) water to a concentration of 100 μM as a storage solution and stored at −80°*C.* Prior to use, it was diluted to 10 μM as a working solution and stored at −20°C.

**Table 3:** The sequences of crRNA.

crRNA	Sequence (5′−3′)
MRV-12a-crRNA1	UAAUUUCUACUAAGUGUAGAUACGCAGACCUGCGCUUUUCC
MRV-12a-crRNA2	UAAUUUCUACUAAGUGUAGAUCCACGCCGUCAAAGCGCUCA
MRV-12a-crRNA3	UAAUUUCUACUAAGUGUAGAUACGGCGUGGGAAAAGCGCAG
MRV-12a-crRNA4	UAAUUUCUACUAAGUGUAGAUUGGUGCAAAACGUCACUCAC
MRV-12a-crRNA5	UAAUUUCUACUAAGUGUAGAUCACCAUAAACAUGAGCGCUU
ISKNV-12a-crRNA1	UAAUUUCUACUAAGUGUAGAUGUGCAUCUGGACCUCAGGUU
ISKNV-12a-crRNA2	UAAUUUCUACUAAGUGUAGAUCAGUAAAGAACGUCACCCAC
ISKNV-12a-crRNA3	UAAUUUCUACUAAGUGUAGAUCUGCAAAGAACAAGGCCUUC
ISKNV-12a-crRNA4	UAAUUUCUACUAAGUGUAGAUCACGUUGCGGUGGGUGACGU

### 2.6 Screening of crRNA

The purified crRNA was used in the CRISPR/Cas12a reaction. The 20 μL reaction mixture comprised 2 μL of 10× HOLMES Buffer 1 (Tolobio, China), 0.5 μL of 10 μM Cas12a protein (EZassay Biotech, China), 0.5 μL of 10 μM crRNA, 2 μL of target DNA, 0.5 μL of 10 μM ssDNA reporter, and 14.5 μL of DEPC water. Viral genomic DNA served as the positive template, while DEPC water acted as the negative template. Primer pairs MRV-RPA-F1/MRV-RPA-R1 and ISKNV-RPA-F3/ISKNV-RPA-R1 were used for the RPA reaction. After a 20-min reaction, the product was directly used as target DNA for the CRISPR/Cas12a reaction without further purification. The CRISPR/Cas12a reaction was conducted in a LineGene9600Plus real-time fluorescent quantitative PCR instrument (BIOER, China) maintained at a constant temperature of 37°C, with fluorescence signals collected every 30 s. Post-reaction observation was performed using a blue light gel documentation system.

### 2.7 Establishment and optimization of the CRISPR/Cas detection system

To enhance the visualization of the Cas12a/crRNA-based detection system, adjustments were made to the ratio of Cas12a protein to crRNA and the concentration of Cas12a protein. Specifically, various combinations of Cas12a protein and crRNA volumes were tested, including 0.1 μL/0.1 μL, 0.1 μL/0.2 μL, 0.25 μL/0.25 μL, 0.25 μL/0.5 μL, 0.5 μL/0.5 μL, and 0.5 μL/1 μL (with both Cas12a protein and crRNA at concentrations of 10 μM). After determining the optimal concentrations of Cas12a protein and crRNA, further optimization was conducted for the amount of 10 μM ssDNA reporter (5′-FAM-TTATT-BHQ1–3′) by testing different volumes of 0.25 μL, 0.5 μL, 1 μL, 1.5 μL, and 2.5 μL.

### 2.8 Detection of RNA targets

RNA targets from MRV and ISKNV were detected using viral RNA as the test template. Positive controls were established with viral DNA, while DEPC water served as the negative control. The RT-RPA reaction was carried out under previously optimized conditions for 30 min, using 5 μL of the un-purified product as target DNA for the CRISPR/Cas12a reaction. All other conditions were maintained consistently with previously determined optimal settings. The reactions were placed in a preheated real-time fluorescent instrument at 37°C, and fluorescence values were recorded every 30 s.

### 2.9 Non-excitation light visual detection

Different fluorophore-quencher pairs, including 5′-FAM-TTATT-BHQ1-3′, 5′-HEX-TTATT-BHQ1-3′, 5′-CY3-TTA TT-BHQ2–3′, 5′-ROX-TTATT-BHQ2-3′, and 5′-CY5-TTATT- BHQ3-3′, were tested to explore the potential for visual detection without excitation light. ISKNV viral DNA was used as the positive template, while DEPC water served as the negative template. The primer pair ISKNV-RPA-F3/ISKNV-RPA-R1 was employed for the RPA reaction, which proceeded for 30 min. The reaction product was directly used as target DNA for the CRISPR/Cas12a reaction without purification. The final probe concentration was set to 10 μM, with all other conditions matching previously optimized settings. Following a 15-min reaction period, color changes were observed with the naked eye to assess the visual detection capability of each fluorophore-quencher pair.

### 2.10 Sensitivity experiment

The RPA-CRISPR/Cas12a reaction was performed using recombinant plasmids containing DNA fragments of the *MRV-MCP* and *ISKNV-MCP* genes at varying concentrations as templates, under the previously mentioned optimal conditions. For the RPA step, 15 μL of the template was added. After a 30-min reaction, 5 μL of the un-purified product was taken directly as the target DNA for the CRISPR/Cas12a reaction. Subsequently, 5 μL of this target DNA was added to the CRISPR/Cas12a reaction mixture. Three replicate wells were prepared for each group of CRISPR/Cas12a reactions and placed in a preheated real-time fluorescent quantitative PCR instrument at 37°C, with fluorescence signals collected every 30 s. Simultaneously, three additional replicate wells were prepared and placed in a 37°C metal bath. These wells were removed at 15 min and 60 min, respectively, for observation under a blue light gel documentation system.

### 2.11 Specificity experiment

In the specificity experiment, nucleic acids from MRV, ISKNV, and SCRV were used as test templates, and DEPC-treated water was used as the blank control. Both the test templates and the blank control were, respectively, added to the RPA reaction mixtures. The RPA-CRISPR/Cas12a detection was conducted using the previously optimized conditions. A real-time fluorescent quantitative PCR instrument was used to monitor the fluorescence values. After 15 min, the samples were removed for observation under a blue light gel documentation system.

### 2.12 Reproducibility experiment

RPA-CRISPR/Cas12a reaction was conducted under previously optimized conditions, using MRV and ISKNV viral DNA as templates, with DEPC water serving as the negative control. RPA amplification reaction was performed in parallel six times, each set for 30 min. From each reaction product, 5 μL was used as target DNA for the CRISPR/Cas12a system, and three replicate wells were set up for each group. A real-time fluorescent quantitative PCR instrument was used to maintain the temperature and monitor the fluorescence intensity. After 15 min, samples were removed and observed under a blue light gel documentation system.

### 2.13 Detection of actual samples

Gut tissue samples were collected from diseased mandarin fish at the Hengxing Aquaculture Farm in Guangzhou, China. Nucleic acids were extracted following the method outlined in Section 2.2. These samples were subsequently tested using our established RPA-CRISPR/Cas12a detection system. Additionally, nested PCR was performed on the samples using the 2× Hieff Ultra-Rapid II HotStart PCR Master Mix (Yeasen, China). The primers employed for the nested PCR are listed in Table 4. If no target bands were observed on electrophoresis following the first-round reaction products with outer primers, a second-round amplification was performed using inner primers.

**Table 4:** Nested PCR primers and sequences.

Primers	Sequence (5′−3′)
MRV-OF (outer primer)	ATGTCTTCTGTTACGGGTTCTGGC
MRV-OR (outer primer)	TTACAGGATGGGGAAACCCATGG
MRV-IF (inner primer)	TTTACCAAACTGCCTACGGCT
MRV-IR (inner primer)	CAACAACAGGAGTGACGCAAG
ISKNV-OF (outer primer)	ATGTCTGCAATCTCAGGTGCAAAC
ISKNV-OR (outer primer)	TTACAGGATAGGGAAGCCTGCG
ISKNV-IF (inner primer)	CCTTAATTTGCCCATTCCCCTCTTC
ISKNV-IR (inner primer)	AGTAGTCTACTCCCATCTGGTGGAG

### 2.14 Data analysis and figure preparation

Data analysis was conducted using GraphPad Prism 9, and all statistical graphs were also produced by the same software. Additionally, schematic diagrams were drawn by Figdraw.

## 3 Results

### 3.1 Screening of RPA primers and crRNA

**Figure 1:**
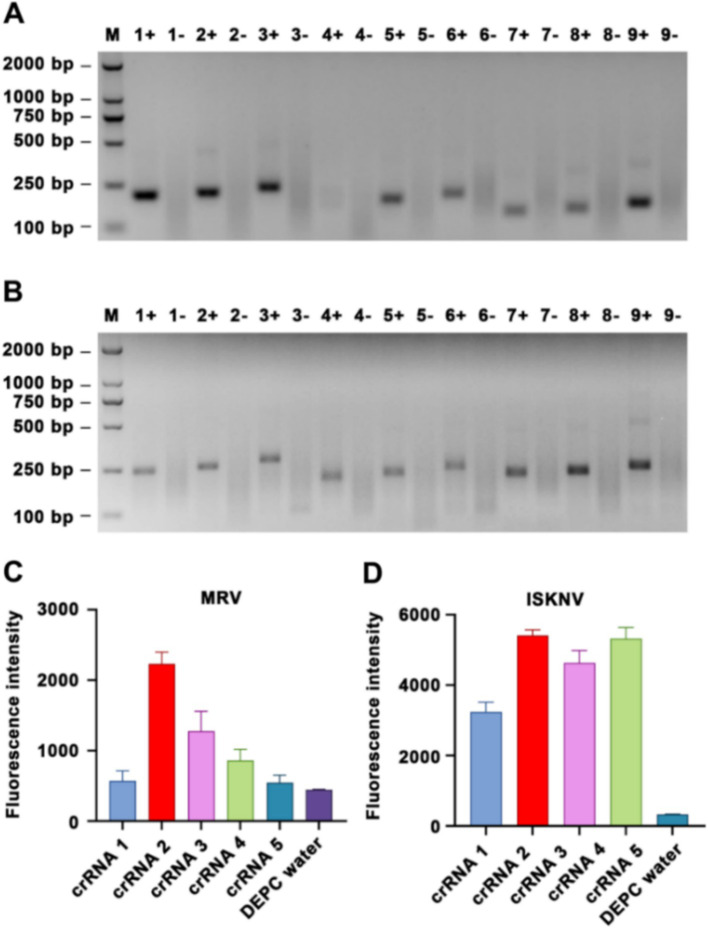
Screening of RPA primers and crRNA. Nucleic acid agarose gel electrophoresis results are presented, showing amplification of **(A)** MRV templates and **(B)** ISKNV templates using various RPA primers. For MRV templates, the following primer pairs were used: 1, MRV-RPA-F1/MRV-RPA-R1; 2, MRV-RPA-F1/MRV-RPA-R2; 3, MRV-RPA-F1/MRV-RPA-R3; 4, MRV-RPA-F2/MRV-RPA-R1; 5, MRV-RPA-F2/MRV-RPA-R2; 6, MRV-RPA-F2/MRV-RPA-R3; 7, MRV-RPA-F3/MRV-RPA-R1; 8, MRV-RPA-F3/MRV-RPA-R2; 9, MRV-RPA-F3/MRV-RPA-R3. “+” represents a positive amplification result, while “−” represents a negative result. Similarly, for ISKNV templates, the primer pairs used were: 1, ISKNV-RPA-F1/ISKNV-RPA-R1; 2, ISKNV-RPA-F1/ISKNV-RPA-R2; 3, ISKNV-RPA-F1/ISKNV-RPA-R3; 4, ISKNV-RPA-F2/ISKNV-RPA-R1; 5, ISKNV-RPA-F2/ISKNV-RPA-R2; 6, ISKNV-RPA-F2/ISKNV-RPA-R3; 7, ISKNV-RPA-F3/ISKNV-RPA-R1; 8, ISKNV-RPA-F3/ISKNV-RPA-R2; 9, ISKNV-RPA-F3/ISKNV-RPA-R3. The detection results for **(C)** MRV and **(D)** ISKNV using various crRNAs are compared. The y-axis indicates the relative fluorescence intensity, while the x-axis indicates the various types of crRNA added to the samples.

To identify the optimal primers, we adhered to the principle of selecting the most challenging reaction conditions and set the RPA reaction time to 20 min. After amplification with the primers, the products were extracted using phenol-chloroform and subjected to electrophoresis. When amplifying the MRV template, all primer pairs, except for pair 4 (MRV-RPA-F2/MRV-RPA-R1), effectively amplified the target fragment; however, they varied in terms of specificity and yield (Figure 1A). Similarly, all primer pairs effectively amplified the ISKNV template (Figure 1B). Based on products specificity and non-specific by-products levels, we selected MRV-RPA-F1/MRV-RPA-R1 and ISKNV-RPA-F3/ISKNV-RPA-R1 as the optimal primer pairs.

Using the optimal primers for each target, we conducted RPA reactions and incorporated the products into the CRISPR/Cas12a system. Different crRNAs were added to the system, and after a 15-min reaction, the highest fluorescence intensities for MRV and ISKNV were observed with MRV-crRNA2 and ISKNV-crRNA2, respectively (Figures 1C,D). Replacing the crRNA with DEPC water resulted in a significant reduction in fluorescence, confirming the effectiveness of the selected crRNAs. Therefore, MRV-crRNA2 and ISKNV-crRNA2 were chosen as the optimal crRNAs for subsequent experiments with MRV and ISKNV.

### 3.2 Optimization of the CRISPR/Cas12a reaction system

**Figure 2:**
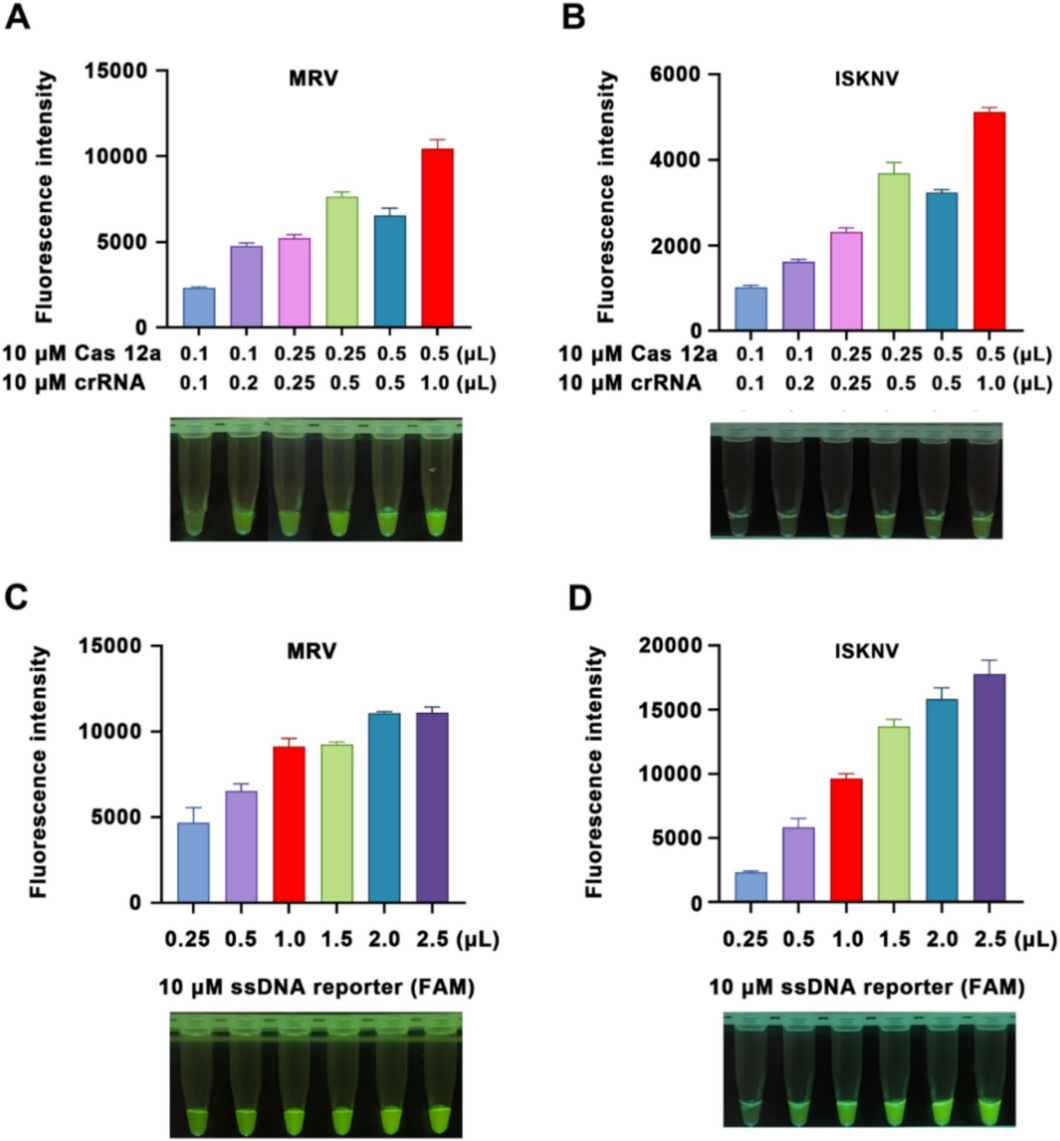
Optimization of the reaction system. **(A,B)** Detection of Cas12a protein and crRNA concentrations for MRV and ISKNV, respectively. **(C,D)** Comparison of detection results using different concentrations of fluorescent probes for MRV and ISKNV, respectively. The x-axis represents the volumes of 10 μM Cas12a protein and crRNA used, while the y-axis indicates the relative fluorescence intensity.

To achieve optimal detection results and minimize costs, we modified the ratios and quantities of Cas12a protein and crRNA. Using viral DNA as the sample, we observed an increase in fluorescence values upon the addition of Cas12a protein and crRNA after a 15-min of CRISPR/Cas12a reaction (Figures 2A,B). The highest fluorescence intensity, with good visual effects, was observed when 0.5 μL of 10 μM Cas12a protein and 1 μL of crRNA were added; this ratio was therefore selected for subsequent experiments. Furthermore, an increase in the amount of fluorescent probe led to a corresponding increase in fluorescence intensity (Figures 2C,D). Considering observations under blue light and cost factors, 1 μL of 10 μM ssDNA reporter was chosen as the optimal amount of fluorescent probe for subsequent experiments.

### 3.3 Capability to detect RNA targets and optimization for use without additional excitation light

**Figure 3:**
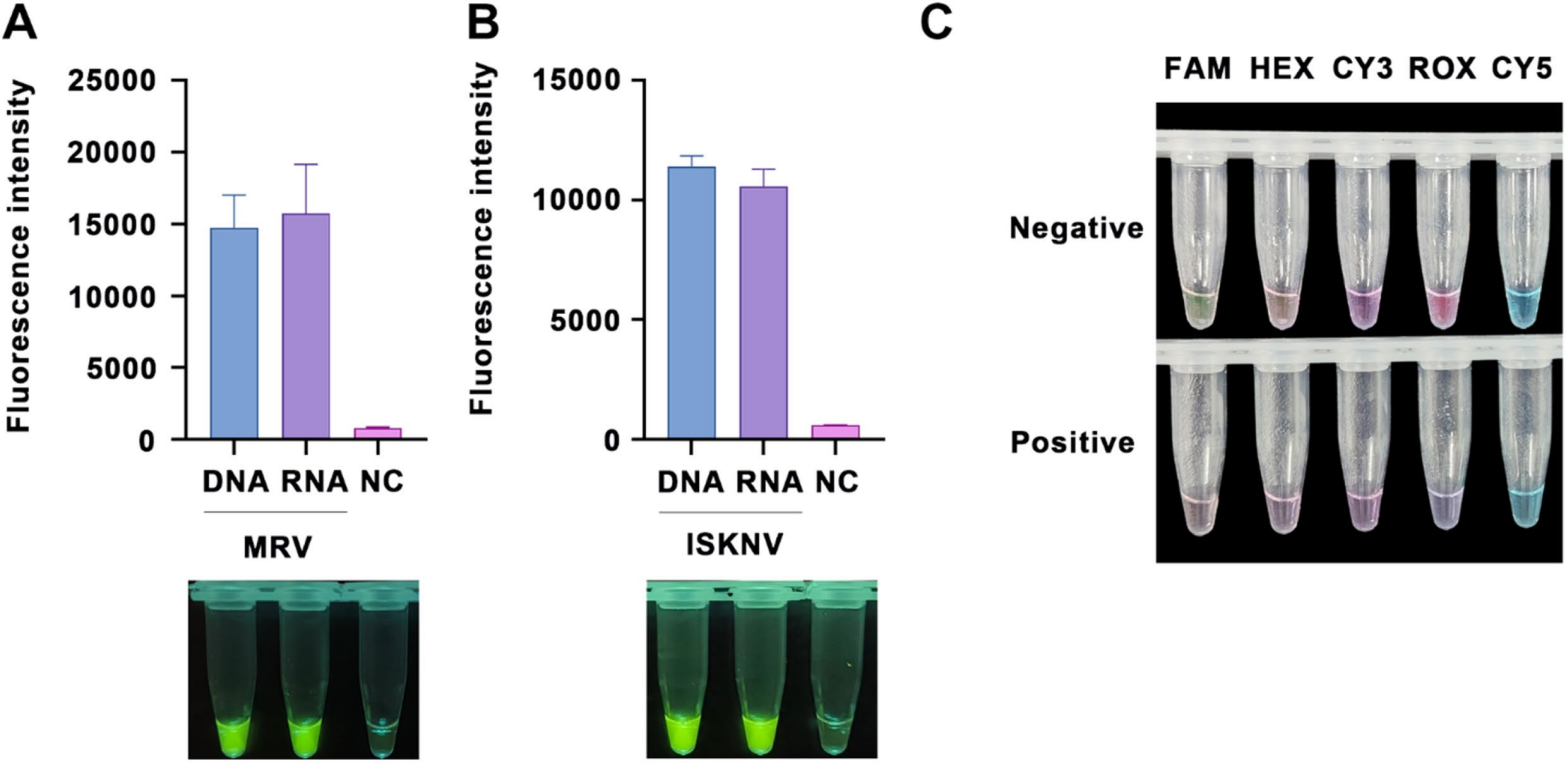
RNA detection capability and probe optimization. Validation of the ability to detect RNA targets (treated with DNase) using **(A)** the MRV detection system and **(B)** the ISKNV detection system. The x-axis represents the types of templates used, and the y-axis represents the relative fluorescence intensity. **(C)** Detection of positive samples using various types of fluorescent probes at a final concentration of 10 μM, observed under natural light.

To enable the detection of RNA targets, reverse transcriptase was incorporated into our RPA amplification system, facilitating reverse transcription by the RPA primers. This capability was validated using virus RNA samples treated with DNase. The results showed that, compared to DNA targets, RNA targets also produced positive fluorescence (Figures 3A,B), clearly distinguishable from the negative controls. Furthermore, we tested the efficacy of five different fluorescently labeled probes. When using high concentrations (final concentration of 10 μM) of the fluorescent probes, probes labeled with FAM or ROX could be visually observed under natural light for positive nucleic acid samples, thus eliminating the need for UV or blue light excitation (Figure 3C).

### 3.4 Evaluation of sensitivity

**Figure 4:**
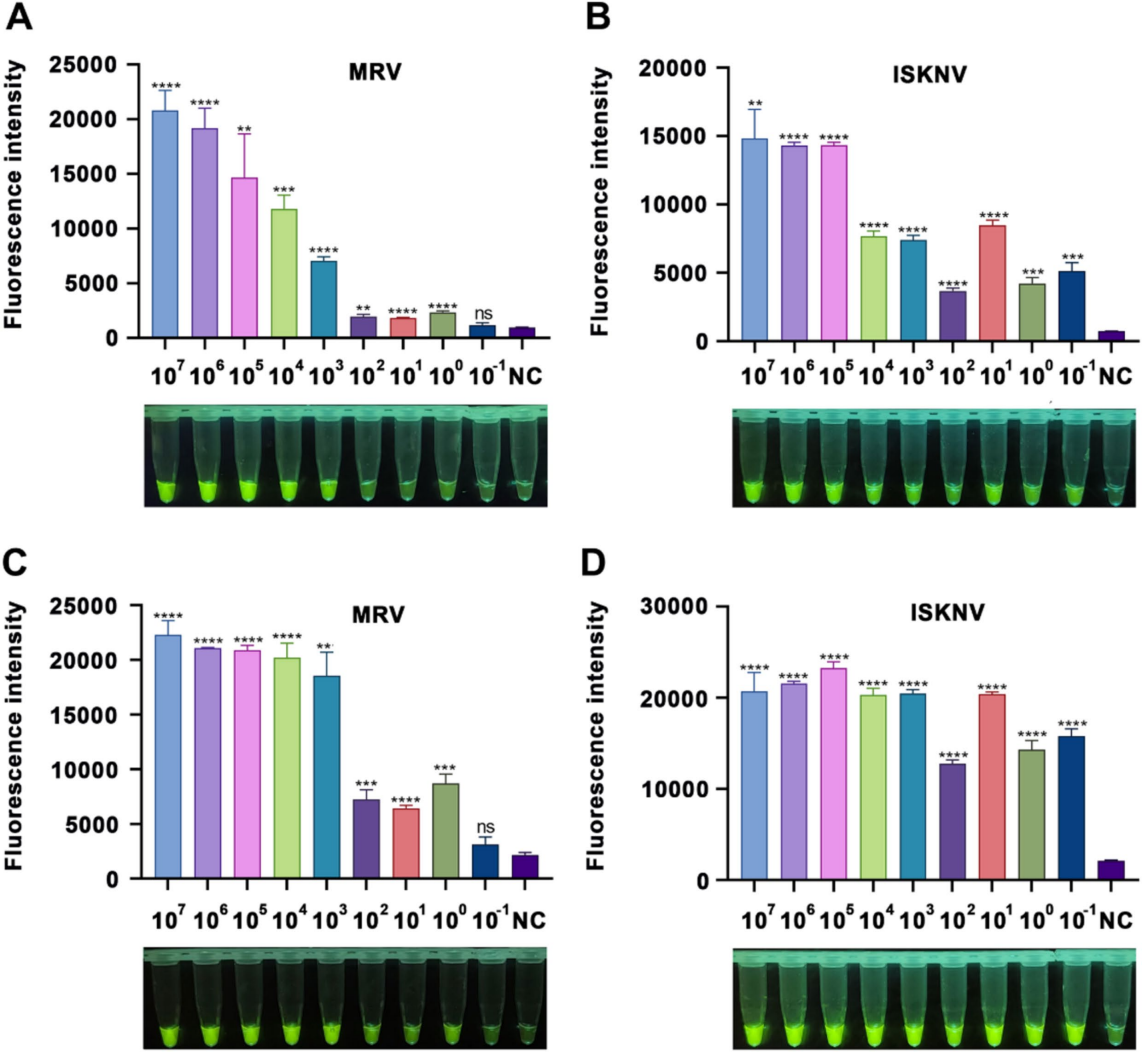
Sensitivity of the CRISPR/Cas12a detection method. **(A,B)** Fluorescence intensity after a 15-minCRISPR/Cas12a reaction with varying concentrations of MRV and ISKNV standards. **(C,D)** Fluorescence intensity after a 60-min CRISPR/Cas12a reaction with different concentrations of MRV and ISKNV standards. The x-axis represents the standard sample concentration, and the y-axis indicates the relative fluorescence intensity. NC indicates DEPC water. Significant differences are indicated by asterisks (*). Ns, not significant (*p* > 0.05); *p < 0.05; **p < 0.01; ***p < 0.001; ****p < 0.0001.

To assess the sensitivity of the RPA-CRISPR/Cas12a detection technology, we performed gradient dilutions of standard samples for the I*SKNV-MCP* and *MRV-MCP* genes, using the previously established optimal conditions for detection. Following the addition of target DNA, notable changes in fluorescence signals were evident (Figure 4).

For *MRV-MCP* gene detection, at a reaction time of 15 min, samples with a concentration of at least 10^3^ copies/μL showed visible fluorescence to the naked eye, while the fluorometric instrument could detect a difference between 1 copy/μL samples and the negative control (Figure 3A). At 60 min, samples with a concentration as low as 1 copy/μL also showed visible fluorescence (Figure 3C). In the case of ISKNV detection, regardless of whether the reaction time was 15 min (Figure 3B) or 60 min (Figure 3D), standard samples with concentrations as low as 10^−1^ copies/μL were detected, and the fluorescence was visible to the naked eye. A prolonged reaction time (60 min) facilitated easier visualization of the fluorescence. These results indicated that our method could detect as low as 1 copy/μL of MRV and 0.1 copy/μL of ISKNV.

### 3.5 Evaluation of specificity

**Figure 5:**
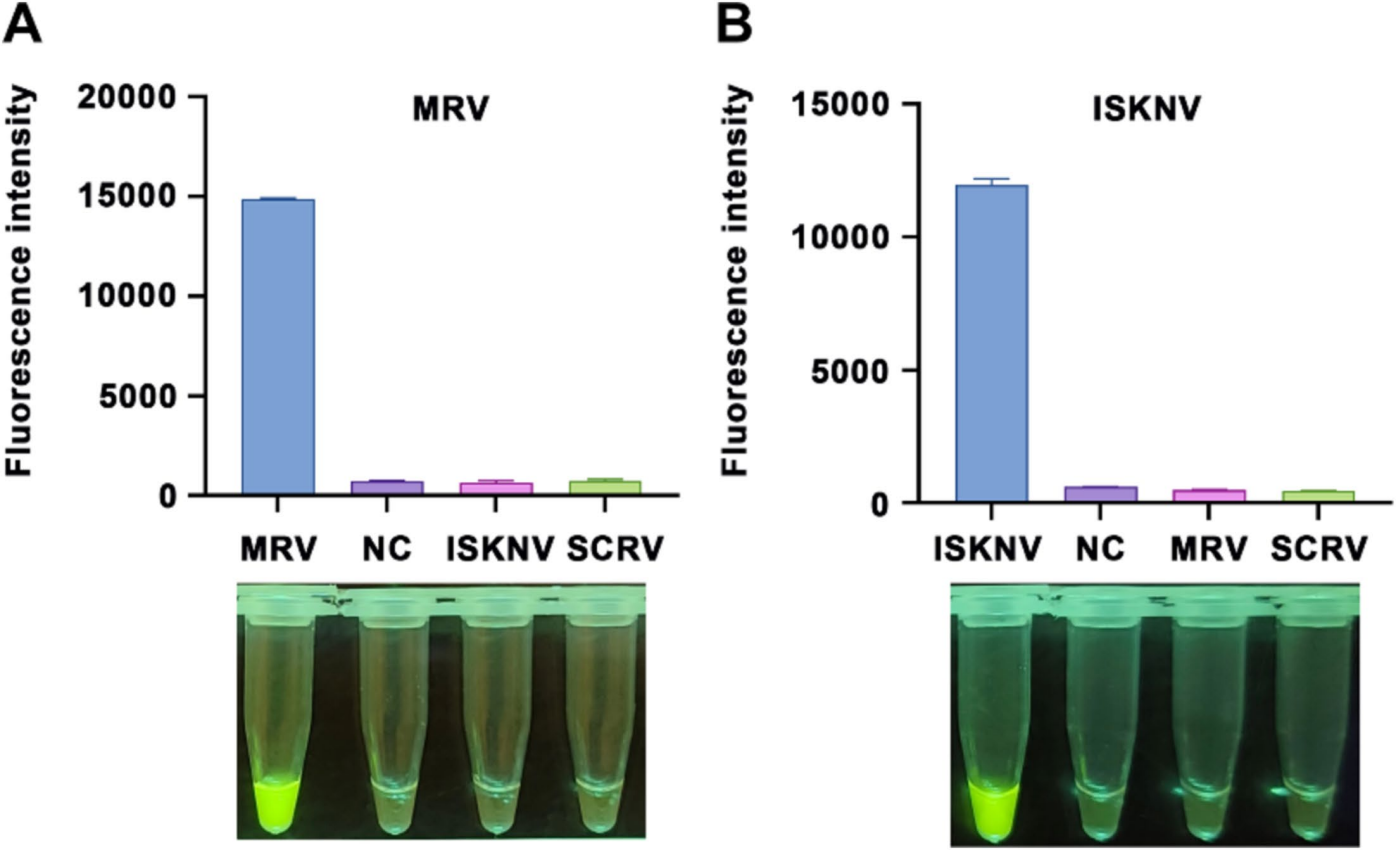
Specificity of the CRISPR/Cas12a detection method. Validation of the specificity for the **(A)** MRV detection system and **(B)** ISKNV detection system using different templates. The *x*-axis represents the types of templates used, and the *y*-axis indicates the relative fluorescence intensity. NC indicates DEPC water.

To verify the specificity of our method, we used multiple viral samples for testing. As shown in Figure 5, the MRV and ISKNV detection systems generated positive signals only when detecting their respective target nucleic acids. When detecting non-target nucleic acids, the fluorescence intensity was comparable to that of the negative control group, indicating no false-positive signals. This confirms the high specificity of our method toward target nucleic acids.

### 3.6 Evaluation of reproducibility

**Figure 6:**
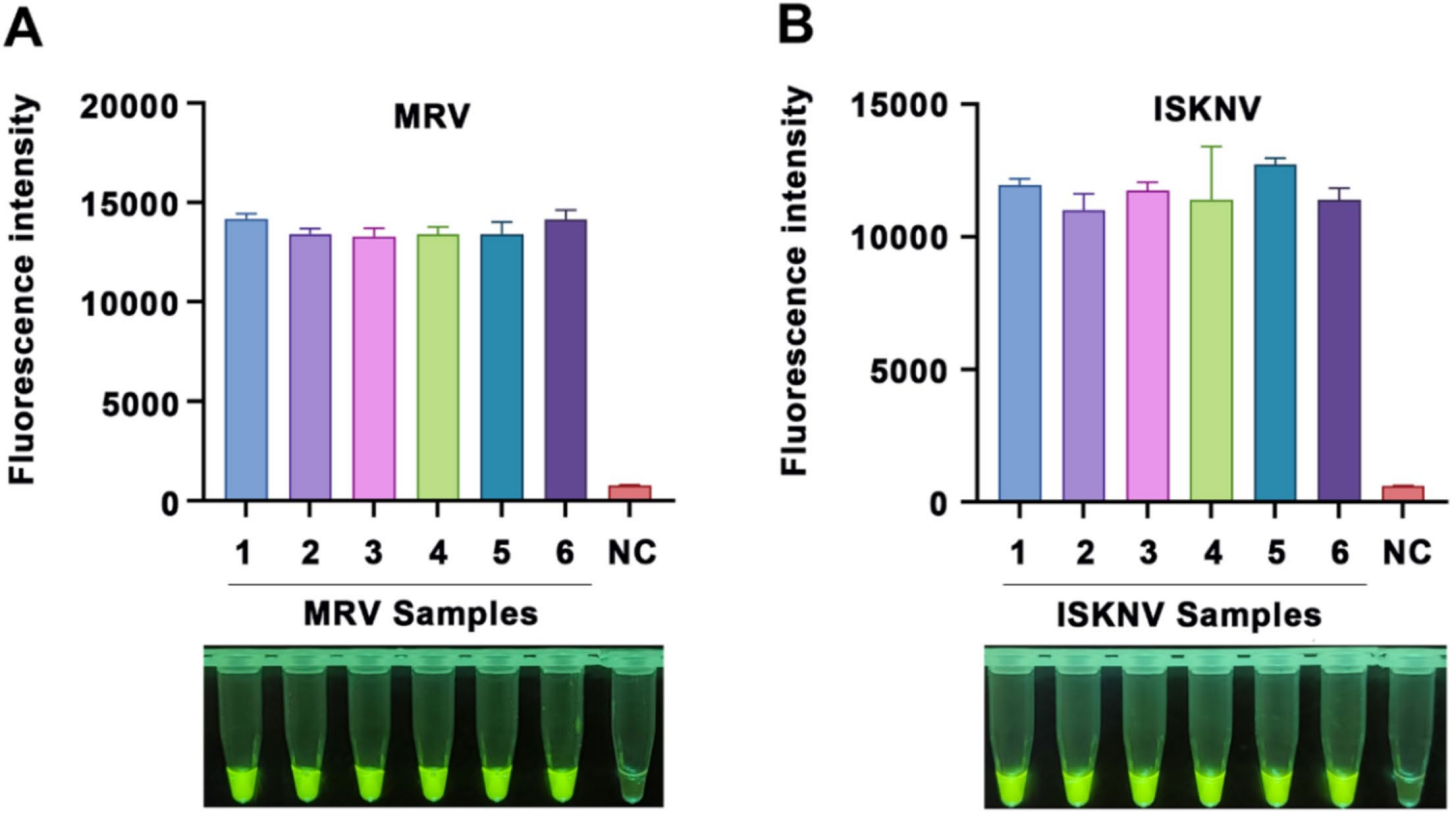
Reproducibility of the CRISPR/Cas12a detection method. Reproducibility validation of the **(A)** MRV detection system and **(B)** ISKNV detection system using positive DNA templates. Each sample was tested six times. The x-axis represents the templates used and their respective replicates, while the y-axis represents the relative fluorescence values, as well as NC indicates DEPC water.

To assess the reproducibility of our detection system, we conducted six independent RPA amplification reactions using MRV and ISKNV positive nucleic acids as templates. The products of these reactions were subsequently analyzed using their respective CRISPR/Cas12a systems. Our findings revealed that the same sample tested positive in all six detections, with minimal variation in fluorescence values (Figure 6). Using the formula variation (CV) = (standard deviation / mean) × 100%, we calculated the coefficient of CV for the MRV detection system (Figure 6A) to be 2.74% and for the ISKNV detection system (Figure 6B) to be 5.12%. These results indicate that the established method has good reproducibility.

### 3.7 Evaluation of the detection capability in tissue samples

**Figure 7:**
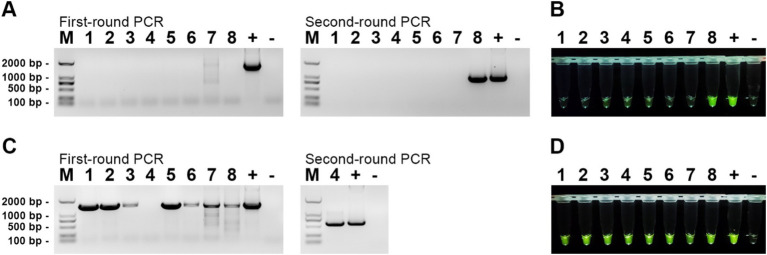
Detection results of actual samples. **(A)** Gel electrophoresis results of nested PCR products for MRV detection in the samples; **(B)** RPA-CRISPR/Cas12a detection results for MRV in the samples; **(C)** Gel electrophoresis results of nested PCR products for ISKNV detection in the samples; **(D)** RPA-CRISPR/Cas12a detection results for ISKNV in the samples. The numbers 1–8 represent the samples, “+” represents the positive control, and “−” represents the negative control.

To evaluate the efficacy of our established RPA-CRISPR/Cas12a detection system, we collected eight gut tissue samples from diseased mandarin fish at the Hengxing Aquaculture Farm in Guangzhou, China. Nucleic acids were extracted from these samples, and both the RPA-CRISPR/Cas12a detection system and nested PCR were employed for analysis. The results were presented in Figure 7. For MRV detection, the first round of nested PCR did not detect any positive, but the second round identified MRV in sample 8 (Figure 7A). Using the RPA-CRISPR/Cas12a detection system, we directly observed strong fluorescence in sample 8, aligning with the nested PCR results (Figure 7B). For ISKNV detection, all samples except sample 4 were positive in the first round of nested PCR. A second round PCR confirmed positivity in sample 4 as well (Figure 7C). Our RPA-CRISPR/Cas12a detection system identified all samples as positive (Figure 7D), mirroring nested PCR results. These results indicated that our detection system performs on par with nested PCR in practical applications, while offering superior time efficiency and convenience.

## 4 Discussion

MRV and ISKNV are widely present in both marine and freshwater fish, exhibiting highly pathogenicity and lethality, resulting in significant economic losses in the aquaculture. In the cultivation of economically valuable fish, disease surveillance becomes critical, as early detection of viral infections allows for swift control measures. Therefore, there is an urgent demand for high-sensitivity, low-cost effective detection methods that can be used on-site. Cell-based methods, which rely on virus isolation, are intricate, time-consuming, and necessitate laboratory facilities (Guo et al., 2022). Currently, molecular biology techniques, such as PCR, quantitative fluorescence PCR (qPCR), and isothermal amplification, are the most prevalent methods (Koda et al., 2023; Zhu et al., 2020). Traditional PCR methods have lower sensitivity, whereas qPCR achieves high sensitivity and specificity (Cao et al., 2024). For example, qPCR can detect ISKNV at a sensitivity of 7 copies/μL (Cao et al., 2024). However, a limitation of qPCR is its requirement for specialized equipment, which may not be available at aquaculture sites (Zhu et al., 2020). The emergence of isothermal amplification technologies, such as Loop-mediated isothermal amplification (LAMP) and RPA, enables nucleic acid amplification without the need for thermal cycling, simplifying the achievement of constant temperature condition compared to thermal cyclers. For aquaculture viruses, LAMP-based detection methods for largemouth bass virus have achieved a sensitivity of 8.55 copies/μL (Zhu et al., 2020), while the recombinase-aided amplification (RAA) system reaches 89 copies/μL (Zhu et al., 2022). Existing LAMP-lateral flow dipstick systems for ISKNV detection have attained a sensitivity of 10 copies/μL (Ding et al., 2010).

CRISPR/Cas-based detection methods, leveraging their trans-cleavage activity’s sensitivity to mismatches (Chen et al., 2018; Abudayyeh et al., 2016), offer high specificity and are widely applied in disease detection. In human disease detection, RPA-CRISPR/Cas-based technologies can detect pseudorabies virus and Japanese encephalitis virus at the attomole (aM) level (Li et al., 2018). For agriculture, He et al. develops a potato virus Y detection system with a sensitivity of 3 × 10^2^ copies/μL, outperforming PCR and qPCR methods (He et al., 2022). Zhang et al. creates a detection system for *Aphelenchoides besseyi*, achieving a minimum detection limit of 1 copy/μL via fluorescence detection and 10^3^ copies/μL via LFA detection (Zhang et al., 2022). In livestock, Mao et al. integrates RPA, CRISPR/Cas12a, and magnetic bead -ssDNA-alkaline phosphatase for rapid, accurate ASFV genes detection, reducing background signals and achieving a high signal-to-noise ratio (Mao et al., 2023). This method allows naked-eye detection of 10 copies/μL ASFV gene (Mao et al., 2023). In aquaculture, Fei et al. establishes an infectious hematopoietic necrosis virus detection system with 9.5 copies/μL sensitivity (Rong et al., 2024), while Thanwarat Sukonta et al. achieves high-sensitivity detection of scale drop disease virus at 40 copies per reaction (Sukonta et al., 2022). RPA-CRISPR/Cas detection system is also applicable for bacterial detection, as demonstrated by Xiao et al.’s *Vibrio vulnificus* detection system with a sensitivity of 2 copies/μL sensitivity (Xiao et al., 2021).

**Figure 8:**
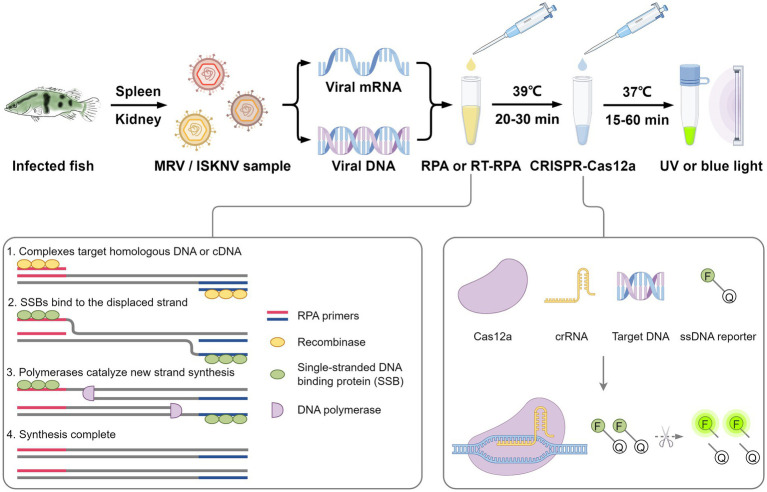
Schematic diagram of the RPA-CRISPR/Cas12a detection method of MRV and ISKNV.

Based on the powerful amplification capability of RPA and the high specificity of the CRISPR/Cas system, we designed and established a detection system for the widely prevalent ISKNV and MRV viruses (Figure 8). To optimize the CRISPR/Cas12a reaction, we systematically evaluated parameters to enhance detection efficiency and specificity, recognizing that optimal Cas protein and crRNA amounts and ratios vary across systems. For example, detecting ASFV requires a Cas12a:crRNA ratio of 1:2 (Mao et al., 2023), while *Pneumocystis jirovecii* detection optimizes at 1:4 (Liu et al., 2024). Similarly, using the CRISPR/Cas system for Red-spotted grouper nervous necrosis virus detection highlights the importance of appropriate Cas protein and crRNA stoichiometry (Huang et al., 2022). Excess Cas protein or crRNA does not guarantee better results, so we optimized the ratio and concentration within cost constraints. We also adjusted fluorescent probe concentrations to maximize the signal-to-noise ratio and minimize background interference. Sensitivity evaluation demonstrated our system could detect as low as 1 copy/μL of MRV and 0.1 copy/μL of ISKNV, indicating its potential for early detection and intervention. Validation with different viral templates and multiple repeat trials confirmed the method’s accuracy, reliability, and reproducibility, and our system also detects viral mRNA by incorporating reverse transcriptase. Optimizing fluorescent probes for natural light detection eliminates the need for specialized equipment, enhancing the method’s accessibility and practicality for field use. Collectively, these optimizations and evaluations contribute to a robust, sensitive, specific, and reproducible detection method for various settings, enabling monitoring and management of viral infections even in resource-limited environments.

Additionally, we have successfully freeze-dried the enzymes and RPA primers required for the RPA reaction system into a single tube, simplifying the reaction process. However, this study does have some limitations. Firstly, nucleic acid extraction and purification from samples remain a prerequisite for detection. In our practice, we tested several commercially available rapid lysis buffers, but the outcomes were unsatisfactory. Researchers in similar fish virus detection studies have opted for extraction methods such as RNAiso Plus (Huang et al., 2022), the virus DNA/RNA extraction kit (Rong et al., 2024), and protease K treatment coupled with phenol-chloroform-isoamyl alcohol extraction (Sukonta et al., 2022). Future advancements in lysis reagents tailored for fish samples and enhancements in the system’s impurity tolerance could greatly expand the technology’s application potential. Secondly, our system entails two sequential reaction steps, which can lead to aerosol contamination when the lid is opened. Due to the system’s unique design, we cannot employ UDG ribonuclease to prevent aerosol contamination, as is done in PCR (Longo et al., 1990). Therefore, developing a one-pot method based on the current system could effectively mitigate aerosol contamination. Nonetheless, challenges such as buffer development, suboptimal PAM site utilization, and the use of phase-separated solutions in one-pot methods can be inconvenient or detrimental to CRISPR reaction efficiency (Hu et al., 2023). Hu et al. introduces the Light-Start CRISPR-Cas12a Reaction with Caged crRNA, which facilitates rapid and sensitive nucleic acid detection. This method involves modifying the crRNA with 6-nitropiperonyloxymethyl-caged thymidine (NPOM-dt) to temporarily inactivate both the crRNA and CRISPR-Cas12a’s trans-cleavage activity, which is then reactivated upon light exposure, resulting in higher sensitivity compared to conventional one-step methods (Hu et al., 2023). In the future, we plan to explore the application of this method in our system to achieve easier operation and reduced contamination.

Overall, we have designed and established a CRISPR/Cas12a-based detection system targeting the widely prevalent ISKNV and MRV viruses. Our method is fast, sensitive, convenient, providing visual results. Utilizing a standard, inexpensive metal bath and a portable UV lamp, results can be easily obtained, eliminating the need for complex equipment. This enables on-site detection, facilitating timely diagnosis and the implementation of appropriate control measures. By applying this scientific research to practical production, significant losses in the aquaculture industry can be reduced.

## Data Availability

The sequencing results of the standard plasmids have been uploaded to the Genbank database and are available at accession numbers PQ655404 and PQ655405. The data supporting the findings of this study are provided within the article.

## Footnotes

^1^ https://ictv.global/taxonomy
